# Superhydrophilic and Underwater Superoleophobic Copper Mesh Coated with Bamboo Cellulose Hydrogel for Efficient Oil/Water Separation

**DOI:** 10.3390/polym16010014

**Published:** 2023-12-19

**Authors:** Yun Peng, Shuang Zhao, Chuanlin Huang, Feifei Deng, Jie Liu, Chunhua Liu, Yibao Li

**Affiliations:** Engineering Research Center of Jiangxi Province for Bamboo-Based Advanced Materials and Biomass Conversion, College of Chemistry and Chemical Engineering, Gannan Normal University, Ganzhou 341000, China; pengyun@buaa.edu.cn (Y.P.); szhao202211@163.com (S.Z.); hcl1119160736@163.com (C.H.); 18385421104@163.com (F.D.); 18943417506@163.com (J.L.)

**Keywords:** wetting, bamboo cellulose, hydrogel coating, oil/water separation

## Abstract

Super-wetting interface materials have shown great potential for applications in oil–water separation. Hydrogel-based materials, in particular, have been extensively studied for separating water from oily wastewater due to their unique hydrophilicity and excellent anti-oil effect. In this study, a superhydrophilic and underwater superoleophobic bamboo cellulose hydrogel-coated mesh was fabricated using a feasible and eco-friendly dip-coating method. The process involved dissolving bamboo cellulose in a green alkaline/urea aqueous solvent system, followed by regeneration in ethanol solvent, without the addition of surface modifiers. The resulting membrane exhibited excellent special wettability, with superhydrophilicity and underwater superoleophobicity, enabling oil–water separation through a gravity-driven “water-removing” mode. The super-wetting composite membrane demonstrated a high separation efficiency of higher than 98% and a permeate flux of up to 9168 L·m^−2^·h^−1^ for numerous oil/water mixtures. It also maintained a separation efficiency of >95% even after 10 cycles of separation, indicating its long-term stability. This study presents a green, simple, cost-effective, and environmentally friendly approach for fabricating superhydrophilic surfaces to achieve oil–water separation. It also highlights the potential of bamboo-based materials in the field of oil–water separation.

## 1. Introduction

The increasing pollution of oily wastewater poses a significant threat to human health and the ecological environment. Separating oil and water remains a global challenge [1,2,3]. Oily wastewater pollution originates from various sources, including oil spill accidents, and industrial and domestic oily wastewater. The composition of oily wastewater is highly complex [4,5,6]. Consequently, the preparation of efficient oil/water separation materials has become a critical focus in treating oily wastewater pollution. Recently, numerous methods have been reported to address this issue, such as sedimentation separation [7], filtration [8,9,10], membrane separation [11,12,13,14], the flotation method [15], adsorption [16,17,18], coagulation [19,20], and chemical solidification [21,22,23]. However, these methods still have drawbacks, including high cost, poor separation efficiency, high energy consumption, and a lack of environmental friendliness, which limit their widespread application. Most reported oil–water separation materials have been modified using low-surface-energy substances containing fluorine (fluoroalkyl silane, FAS) [24,25]. Additionally, various chemical polymers/materials with non-biodegradability, such as polyurethane foam [26,27], polytetrafluoroethylene [28,29], and polydimethylsiloxane [30,31,32], have been selected as substrate materials. Therefore, there is an urgent need to design and develop cost-effective and environmentally friendly super-wetting separation materials, as well as simple and efficient separation methods, to address the serious problem of oily wastewater pollution.

Cellulose is a linear polymer made of β (1–4)-d-glucose unit, which is primarily produced by plants (such as cotton, wood, straw, and bamboo), algae, tunicates, and some bacteria, serving as a structural polymer [33]. Due to its unique properties, such as availability in large quantities, low costs, biocompatibility, biodegradability and good chemical stability, cellulose also finds applications in fields such as biomedicine, tissue engineering and biomimetic materials [34,35,36,37]. Furthermore, a large number of hydroxyl groups are contained in cellulose, resulting in easy modification and the creation of active sites for fabricating super-wettability surfaces in oil/water separation materials. Therefore, cellulose stands out as the most favorable material for designing and preparing eco-friendly super-wetting oil/water separation materials. With the rapid development of design ideas and fabrication technologies, a significant number of cellulose-based oil/water separation materials have emerged [38,39,40,41,42,43,44,45,46,47,48,49]. For example, Ma et al. reported the successful development of cellulose-coated cotton fabric with hydrophilic and underwater oleophobic properties. This fabrication process involved a simple step of cellulose dissolution and regeneration, resulting in a remarkable capability to separate a wide range of highly emulsified oil–water mixtures with an excellent separation efficiency (>93.2%) and reasonable flux (>4000 L·m^−2^·h^−1^) [50]. The CNF-PDMS (cellulose nanofiber-polydimethylsiloxane) aerogel sheets with superhydrophobic, elastic, and anisotropic characteristics were prepared using a facile method that combined directional freeze-drying, soaking in a polydimethylsiloxane (PDMS) solution, and heat treatment. This method achieved continuous oil/water separation through filtration, with a flux of up to 2800 L·m^−2^·h^−1^ and a separation efficiency of 99.9% [51]. Li and colleagues constructed a novel carbon aerogel composed of natural microfibrils/regenerated cellulose by dissolving economical hardwood pulps in a solvent consisting of a deep eutectic solvent (DES) and NMMO·H_2_O. This carbon aerogel exhibited high compressibility, outstanding elasticity, excellent fatigue resistance, good separation performance, and recyclability [52]. Su and co-authors reported an anisotropic cellulose nanocrystalline sponge sheet using a facile and straightforward fabrication method. This sponge sheet exhibited ultrahigh water fluxes (100,000 L·m^−2^·h^−1^) and oil/water selectivity (up to 99.99%) [53]. While previous reports focused on cellulose derived from hardwood pulps, cotton, and cellulose nanocrystalline, there is relatively little research on bamboo cellulose. Therefore, the development and utilization of super-wetting materials made from bamboo cellulose hold great significance. Bamboo cellulose (BC) originates from natural bamboo and possesses characteristics such as high strength, excellent breathability, good mechanical properties, and biodegradability [54,55,56,57,58,59]. Peng and co-authors developed a method to prepare bamboo cellulose with different levels of Mη through one-step oxidation based on natural BC. This development has the potential to contribute to the advancement and utilization of functional materials made from bamboo cellulose [60]. Additionally, they successfully fabricated a super-hydrophobic/superoleophilic BC foam using a versatile method of surface hydrophobic modification for oil/water separation [41]. However, the oil/water separation process of cellulose-based foam with super-wetting is complex and discontinuous, which hinders its practical application in the field of oil/water separation. Therefore, it is important to develop BC-based materials for “water removing” in oil/water separation to address the issue of oily wastewater. In this study, a super-wetting membrane for "efficient oil/water separation was fabricated using a feasible and environmentally friendly dip-coating method, based on renewable bamboo cellulose. The resulting membrane exhibited superhydrophilicity and underwater superoleophobicity, making it a promising candidate for the treatment of oily wastewater. The surface hierarchical structures and chemical composition of the super-wetting membrane were analyzed using modern techniques. Moreover, the oil/water separation performance of the super-wetting membrane was thoroughly studied through a series of experiments involving the separation of oil/water mixtures. Additionally, the membrane’s high recyclability was demonstrated through a 10-cycle oil/water separation experiment. Furthermore, the mechanism behind the oil/water separation, which involves a gravity-driven “water-removing” mode facilitated by the BC hydrogel-coated super-wetting membrane, was investigated. This research is significant as it offers a green, simple, cost-effective, and environmentally friendly approach to producing a range of super-wetting surface materials to address the issue of oily wastewater pollution. Moreover, it has the potential to enhance the practical application of BC-based materials with super-wetting in the field of oily wastewater treatment.

## 2. Materials and Methods

### 2.1. Materials

Bamboo bleached pulp with a molecular weight of 1.9 × 10^5^–2.2 × 10^5^ α-cellulose content (88%) was supplied by Ganzhou Hwagain Co., Ltd. (Ganzhou, China). Copper meshes with different mesh sizes (100 N, 120 N, 150 N, 180 N and 200 N, the diameters of copper wires are 0.112 mm, 0.081 mm, 0.061 mm, 0.051 mm and 0.051 mm, respectively; the mesh average pore size are 0.152 mm, 0.131 mm, 0.108 mm, 0.090 mm and 0.076 mm, respectively) were purchased from a local hardware supermarket. Lithium hydroxide monohydrate (LiOH·H_2_O, 99.0%) and liquid paraffin (AR) were offered by Shanghai Macklin Biochemical Co., Ltd. (Shanghai, China) Urea (99.0%, AR), and acetone (CH_3_COCH_3_, 99.0%, AR) was provided by Xilong Chemical Co., Ltd. (Beijing, China). Oil Red was purchased from Bio-lab Technology Co., Ltd. (Beijing, China), and 1,2-dichloroethane (C_2_H_4_Cl_2_, 99%, AR), hexane (97%, AR), benzene (C_6_H_6_, 99.5%, AR), toluene (C_7_H_8_, 99.5%, AR), para-xylene (C_8_H_10_, 99.5%, AR) were obtained from Shanghai Aladdin Bio–Chem Technology Corporation (Shanghai, China). Kerosene was purchased from Sinopec SenMei (Fujian) Petroleum Co., Ltd. (Fuzhou, China) Petroleum ether (AR) and ethanol (C_2_H_5_OH, 99.7%, AR) were obtained from Guangdong Xinghua Technology Co., Ltd. (Guangdong, China). Vacuum pump oil was purchased from the Beijing Sifang Special Oil Factory (API: CS 100, Beijing, China). Peanut oil was obtained from the Yimengshan Peanut Oil Co., Ltd. (content of unsaturated fatty acid: 80%, Heze, Shandong, China). Laboratory-made deionized water was used for all experiments and tests. All reagents were used as-received without further purification.

### 2.2. Fabrication of the Superhydrophilic/Superoleophobic Bamboo Cellulose Hydrogel-Coated Copper Mesh

**Figure 1:**
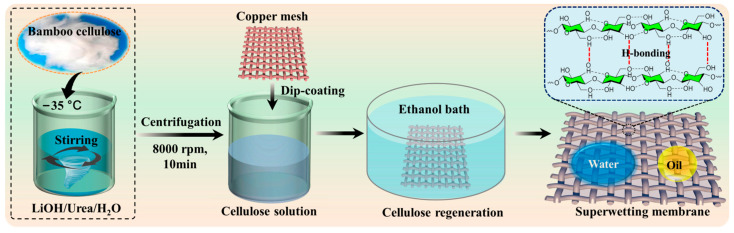
Schematic diagram of fabrication of the super-wetting membrane coated 1 wt% BC hydrogel on copper mesh substrate.

The bamboo pulp was dried at 105 °C for 4 h and then crushed using a wall crusher to obtain fluffy bamboo cellulose. A solution of LiOH·H_2_O/urea/deionized water (8:15:77 by weight) weighing 99 g was pre-cooled to −12.6 °C. Next, 1 g of dried cellulose sample was quickly added to the solvent system and vigorously stirred for 2–5 min, resulting in a uniform and transparent BC solution. The solution was then subjected to high-speed centrifugation at 8000 rpm and 5 °C for 10 min. After that, a pretreated copper mesh measuring 3 cm in length was immersed in a 1 wt% BC solution, causing a layer of BC solution to adhere to the surface of the copper wire on the mesh. The copper meshes coated with the BC solution were then placed in a 75% ethanol coagulation bath for 24 h, resulting in the fabrication of the BC hydrogel-coated copper mesh through the physical regeneration of the BC solution in the alcohol bath. The prepared membrane was washed with a large amount of deionized water to neutralize it and stored in water at room temperature for further testing and characterization. The schematic diagram of the preparation process of the superhydrophilic and underwater superoleophobic BC/copper mesh is shown in Figure 1.

### 2.3. Characterization

The surface of hierarchical structures and elements of the super-wetting cellulose/copper mesh composite membranes were observed by scanning electron microscopy (SEM, Quanta−450 FEI, Gravenhage, The Netherlands) configured with energy dispersive X-ray spectroscopy (EDS), operated at 5.0 kV with sputtered Au, respectively. The surface chemical composition was performed, Fourier transform infrared (FT-IR) spectra were obtained by using a Nicolet FT-IR 5700 spectrophotometer (Nicolet, Madison, Wisconsin, USA) with the KBr pellet method, and we recorded over the range of 4000–400 cm^−1^ at room temperature. The crystal peaks of the bamboo cellulose and the super-wetting cellulose/mesh membranes were determined using an X-ray diffractometer for polycrystalline material investigation (D8, ADVANCE, Karlsruhe, Baden-Württemberg, Germany) with the conditions of the voltage, current, scanning range, and scanning rate being 40 kV, 40 mA, 2*θ* = 5–40°, and 5°/min, respectively. The wettability of the prepared super-wetting membrane surface was measured with a 4 µL droplet using a contact angle analyzer (DSA100, KRÜSS, Hamburg, Germany) at an ambient temperature, and the contact angle result represents the average of the contact angles of five randomly selected positions on the super-wetting membrane surface. The water contact angle in the air and the oil contact angle in the water of the super-wetting membrane were measured, including the tested oil (toluene, petroleum ether, benzene, hexane, para-xylene, pump oil, paraffin liquid, peanut oil, and 1,2-dichloroethane). The optical images and the process of oil–water separation were captured with a digital camera (D7100, Nikon, Tokyo, Japan).

### 2.4. Oil/Water Separation Experiments of Super-Wetting Membrane

A simple oil/water separation device was prepared by combining the prepared superhydrophilic and underwater superoleophobic cellulose hydrogel-coated copper mesh (200 meshes) with a tetrafluoroethylene (PTFE) tube sleeve. Then, 20 mL of organic solvents was dyed red with 2 mg Oil Red. A 40 mL oil/water mixture (*v*/*v*, 1:1) was directly poured into the upper tube of the oil/water separation device, and the oil/water separation was achieved through a “water-removing” mode driven by gravity. A series of oils, including kerosene, toluene, n-hexane, benzene, petroleum ether, and peanut oil, were used and dyed by Oil Red for easy observation. During the separation process, the water was collected after infiltrating the super-wetting membrane, and the oil was excluded above the super-wetting membrane. Then, the oil–water separation efficiency was calculated by measuring the mass of collected water using a high-precision electronic analytical balance (225D-1CN, Sartorius, Gottingen, Germany). The oil/water separation efficiency (*η*) was obtained using Equation (1), and the water flux (*F*) was calculated by Formula (2).

(1) η=m1m2

where *m*_1_ and *m*_2_ are the oil weight before and after the oil/water separation, respectively.

(2) Flux=VST

where *V* is the volume of collected water; *S* is the effective area of the oil/water separation membrane; and *T* is the time required to complete an oil/water separation experiment.

Moreover, the oil intrusion pressure of the BC hydrogel coating super-wetting membrane was investigated. A certain volume of oil (hexane) was slowly added into the oil/water separation device, and just before the oil penetrated the super-wetting membrane, the maximum height of the oil column that the super-wetting membrane supported was observed and measured. Therefore, the maximum intrusion pressure (P) was calculated according to the Equation (3):

(3) $$P=\rho gh_{max}$$

where *P* is as previously mentioned, *ρ* is the density of the oil, g is the gravitational acceleration and *h*_max_ is the maximum height of the oil column supported by the super-wetting membrane.

## 3. Results and Discussion

### 3.1. Micromorphologies, Chemical Compositions and Wettability of the Super-Wetting Membrane

**Figure 2:**
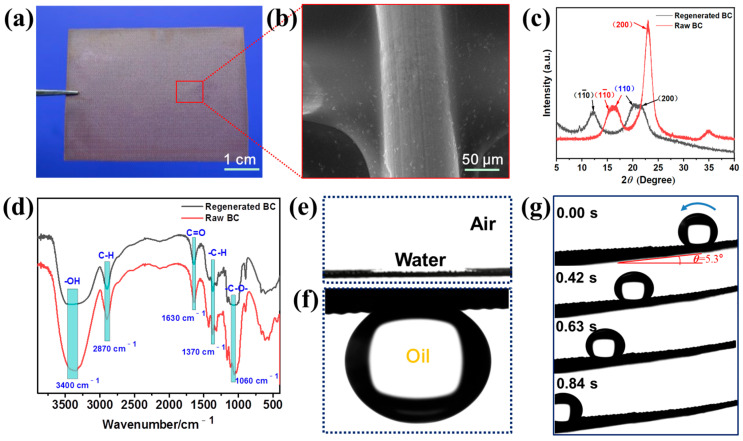
(**a**) The optical image of the super-wetting membrane. (**b**) SEM image of the super-wetting membrane. (**c**) XRD data of the raw BC (red line) and regenerated BC (black line). (**d**) FTIR data of the raw BC (red line) and regenerated BC (black line). Optical photographs of contact angle in air (**e**) and underwater oil (hexane) contact angle (**f**). (**g**) Low adhesion behavior of underwater oil droplet (1,2-dichloroethane) on the super-wetting membrane.

An optical image of the BC hydrogel-coated copper mesh is shown in Figure 2a. The transparent coating of BC hydrogel on the surface of the super-wetting membrane was clearly observed, which was attached to the surface of the copper mesh. As illustrated in Figure 2b, an SEM image of the super-wetting membrane, the BC hydrogel coated on the copper wire surface (Figure S2), especially the cross-connection position of the copper mesh, was covered with a large amount of cellulose hydrogel, and the surface roughness of the copper mesh was enhanced. The EDS data contained three elemental components: C, N, and O, indicating that a bamboo cellulose coating had been formed on the surface of the copper mesh (Figure S3). Moreover, the chemical compositions on the surface of the super-wetting membrane were analyzed by XRD and FTIR spectra. As shown in Figure 2c, the XRD spectrum exhibited raw bamboo cellulose (raw BC) and regenerated bamboo cellulose (regenerated BC). The XRD patterns of the raw BC showed three characteristic peaks at 2*θ* = 14.9°, 16.2° and 22.6° corresponding to the (1$\bar{1}$0), (110), and (200) planes of cellulose I crystalline forms, respectively. However, The XRD patterns of regenerated BC show three characteristic peaks at 2*θ* = 12.1°, 19.7° and 22.0° corresponding to the (1$\bar{1}$0), (110), and (200) planes. These three crystal planes belong to cellulose II. The presence of XRD peaks characteristic of cellulose II for cellulose dissolved in the LiOH·H_2_O/urea/water system indicates the fact that the cellulose structure was completely transformed from cellulose I to cellulose II after the regeneration process. The formation of the BC hydrogel was realized through a physical process by establishing hydrogen bonding (H-bonding) between the cellulose chains. Similarly, the FTIR spectra of the raw BC and regenerated BC certified that the characteristic peaks of cellulose functional groups have not changed either (Figure 2d), demonstrating that the formation of regenerated BC from raw BC is a physical change process. The characteristic peaks at 3400 cm^−1^ correspond to the O-H stretching vibration, which is the characteristic peak of cellulose. The characteristic peaks at 2870 cm^−1^, 1630 cm^−1^, 1370 cm^−1,^ and 1060 cm^−1^ correspond to the C–H stretching vibration, C=O stretching vibration, –C–H stretching vibration and –C–O– stretching vibration, respectively. Furthermore, the surface wettability of the super-wetting membrane coated with the BC hydrogel coating was investigated. The prepared BC hydrogel-coated copper mesh exhibited a special wettability; it is superhydrophilic in the air and superoleophobic in water. An oil droplet (1,2-dichloroethane) remained spherical on the surface of the super-wetting membrane (Figure S1), and the static contact angle of a water droplet (4 µL) in the air is approximately 0° (Figure 2e), while the contact angle of an underwater oil (4 µL, hexane) contact angle is 151° (Figure 2f). The BC hydrogel coating of the prepared super-wetting membrane not only increased the roughness of the copper mesh substrate surface but the hydrophilic hydroxyl groups contained in the BC hydrogel coating also enhanced the hydrophilicity of the copper mesh surface. Moreover, the super-wetting membrane has excellent underwater superoleophobicity and low adhesion—a 4 µL oil droplet (1,2-dichloroethane) quickly rolled off the surface with a tilting angle of 5.3° within 0.84 s (Figure 2g and Movie S3), resulting from the presence of micro/nanoscale structures of the hydrogel-coated super-wetting membrane that are filled with water in aquatic environments.

### 3.2. Performances of Oil/Water Separation

**Figure 3:**
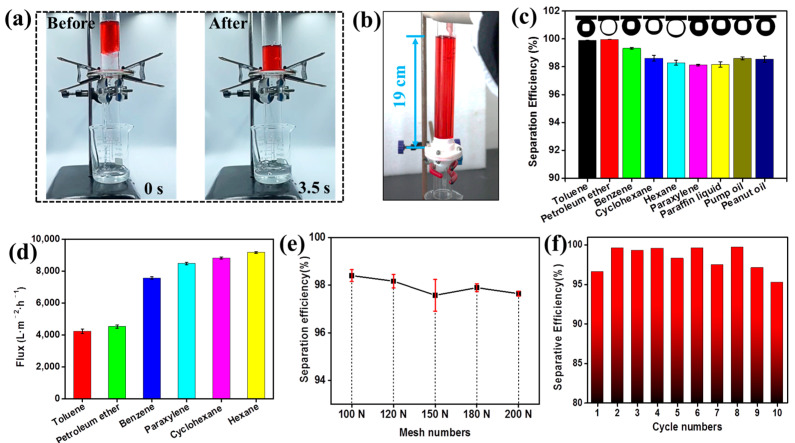
Oil/water separation of the super-wetting membrane. (**a**) Optical images of before/after oil/water separation; (**b**) Optical image of the oil intrusion pressure measurement. (**c**) The oil/water separation efficiency of various oil–water mixtures and underwater contact angles (insert pictures). (**d**) The fluxes of the super-wetting membrane for oil–water mixtures. (**e**) The separation efficiency of the super-wetting membrane with different mesh numbers. (**f**) The relationship between the recycle numbers and separation efficiency.

The comprehensive performance of oil/water separation using a “water-removing”-type super-wetting membrane (1 wt% BC hydrogel) was systematically investigated. Optical images before and after the separation process are shown in Figure 3a. In the mixture solution of water and dyed red toluene, the water component was quickly separated by gravity within 3.5 s, while the oil component was blocked on the surface of the super-wetting membrane. This blocking occurred because the BC hydrogel coating on the membrane surface was filled with water, creating a water/oil interface that hindered the penetration of oil components. In Figure 3b, it can be observed that the maximum height (*h*max) of the oil column (hexane, Movie S2) supported by the super-wetting membrane is about 19 cm, resulting in an oil intrusion pressure of up to 1.23 kPa. This high intrusion pressure is conducive to efficient oil–water separation under gravity-driven conditions. Furthermore, various oil–water mixtures, including toluene, petroleum ether, benzene, cyclohexane, hexane, paraxylene, paraffin liquid, pump oil, and peanut oil, could be successfully separated using the BC hydrogel coating super-wetting membrane driven by gravity (Movie S1), as shown in Figure 3c. The oil/water separation efficiencies for these various oils (organic solvents) were all greater than 98%. Specifically, the oil/water separation efficiencies were 99.89%, 99.96%, 99.32%, 98.61%, 98.28%, 98.12%, 98.16%, 98.61%, and 98.53% for toluene, petroleum ether, benzene, cyclohexane, hexane, paraxylene, paraffin liquid, pump oil, and peanut oil, respectively. The results showed that the super-wetting membrane modified with BC hydrogel had excellent oil–water separation efficiency. Furthermore, the water flux of six kinds of mixed solutions during the oil/water separation process was researched using the super-wetting membrane (200 N copper mesh), as shown in Figure 3d. The water flux was 4326 L/m^2^·h, 4528 L/m^2^·h, 7570 L/m^2^·h, 8485 L/m^2^·h, 8827 L/m^2^·h and 9168 L/m^2^·h for toluene, petroleum ether, benzene, paraxylene, cyclohexane and hexane. The results demonstrated that the super-wetting membrane modified with BC hydrogel could achieve rapid oil/water separation. Moreover, the oil/water separation efficiency of the super-wetting membrane coated with 1 wt% BC hydrogel on a copper mesh substrate with different mesh numbers has been thoroughly explored. The results indicated that the mesh number of the copper mesh substrate had little effect on the oil/water separation efficiency of the super-wetting membrane, as shown in Figure 3e. However, as the number of copper meshes increased, the water flux decreased, which was due to the increase in mass transfer resistance caused by the decrease in the pore size of the copper mesh. Furthermore, the stability performance of the super-wetting membranes was studied by separating a mixed solution (hexane and water). The super-wetting membranes still maintained a high oil–water separation efficiency of up to 95% after 10 cycles, indicating that the BC hydrogel-coated super-wetting membrane had outstanding stability during oil/water separation.

### 3.3. Oil/Water Separation Mechanism of the Super-Wetting Membrane

**Figure 4:**
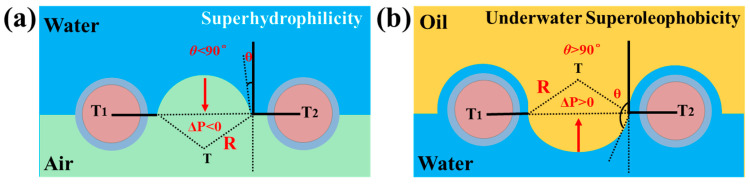
Schematic illustration of the liquid-wetting models. (**a**) Water penetrated the super-wetting membrane in air; (**b**) Oil intercepted the super-wetting membrane surface.

The oil/water separation mechanism of the BC hydrogel-coated super-wetting membrane has been studied, and the liquid-wetting processes are illustrated in Figure 4. The intrusion pressure (ΔP) of the super-wetting membrane could be described by Equation (4): [61]

(4) $$\Delta P=\frac{2\gamma }{R}=\frac{-l\gamma \left(\cos\theta \right)}{A}$$

where *γ* is the interfacial tension, *R* is the meniscus’s radius, *l* is the mesh pore’s perimeter, *A* is the pore’s area, and *θ* is the contact angle on the film. According to Equation (4), the membrane surface was subjected to additional pressure (Δ*P* > 0) as the contact angle was *θ* > 90°. In contrast, the contact angle was *θ* < 90°, resulting in the Δ*P* < 0 and the liquid passing through the super-wetting membrane. Likewise, in the aquatic environment, the micro-/nanoscale structures and the BC coatings of the super-wetting membrane were filled with water, resulting in the formation of a layer of water film on the surface of the super-wetting membrane and strengthening the repulsive force between polar (water) and non-polar (oil) molecules simultaneously. Therefore, the water component was able to pass through the super-wetting membrane, while the water contacted the BC hydrogel coating, owing to the contact angle *θ* being nearly 0° and Δ*P* < 0 (Figure 4a). On the contrary, the oil component was blocked on the super-wetting membrane as the oil contacted the BC hydrogel coating, because of the presence of the water layer, oil was prevented from permeating the super-wetting membrane surface, and *θ* is obviously larger than 90° and Δ*P* > 0 (Figure 4b). According to the above analysis, the prepared super-wetting membrane may be able to achieve efficient oil–water separation.

## 4. Conclusions

In summary, the BC-coated membrane was fabricated using a green and feasible method that involved green dissolution, dip-coating, and regeneration processes. The resulting composite membrane exhibits unique properties of superhydrophilicity (water contact angle, WCA, ~0°) and underwater superoleophobicity (oil contact angle, OCA, ~151°). This super-wetting membrane has the capability to effectively separate oil and water through a process known as “water-removing”, where it can retain oil while allowing water to pass through under gravity. The separation efficiency of the prepared super-wetting membrane is above 98% for various oil/water mixtures, reaching up to 99.96%, with a permeate flux of up to 9168 L·m^−2^·h^−1^. These results demonstrate the efficient oil/water separation performance of the membrane. Additionally, the membrane maintains a separation efficiency of over 95% even after 10 cycles of separation, indicating its excellent long-term stability. This study highlights the potential of using bamboo cellulose, which is cost-effective, biodegradable, and readily available, in the treatment of industrial oily wastewater.

## Data Availability

The data presented in this study are available on request from the corresponding author.

## Supplementary Materials

The following supporting information can be downloaded at: https://www.mdpi.com/article/10.3390/polym16010014/s1, Figure S1: Optical image of an oil droplet (1,2-dichloroethane, dyed by Oil Red O) on the BC hydrogel-coated super-wetting membrane surface; Figure S2: Cross-section SEM images of the BC hydrogel-coated super-wetting membrane; Figure S3: The EDS and elemental analysis of the BC hydrogel-coated super-wetting membrane, (a) the chart of element relative content, (b) images of surface element distribution; Movie S1: Recording the water/hexane (dyed by Oil Red O) separation process of the BC hydrogel-coated super-wetting membrane through a camera; Movie S2: Recording the measuring process of the intrusion pressure of the hexane (dyed by Oil Red O) on the BC hydrogel-coated super-wetting membrane by a camera; Movie S3: Recording the rapid departure process of an oil droplet (1,2-dichloroethane) on the BC hydrogel-coated super-wetting membrane surface with a tilt angle of 5.3° through a camera.
