# Supplementary material for: Superhydrophilic and Underwater Superoleophobic Copper Mesh Coated with Bamboo Cellulose Hydrogel for Efficient Oil/Water Separation
Source: Polymers (Basel). 2023 Dec 19;16(1):14. doi: 10.3390/polym16010014 (PMC10780632; doi:10.3390/polym16010014)
Supplement: Supplementary file 1 [file polymers-16-00014-s001.zip › Supporting information.pdf]

Supplementary Materials

Superhydrophilic and Underwater Superoleophobic Copper Mesh Coated with Bamboo Cellulose Hydrogel for Efficient Oil/Water Separation

Yun Peng, Shuang Zhao, Chuanlin Huang, Feifei Deng, Jie Liu, Chunhua Liu * and Yibao Li *

Engineering Research Center of Jiangxi Province for Bamboo-Based Advanced Materials and Biomass Conversion, College of Chemistry and Chemical Engineering, Gannan Normal University, Ganzhou 341000, China; pengyun@buaa.edu.cn (Y.P.); szhao202211@163.com (S.Z.); hcl1119160736@163.com (C.H.); 18385421104@163.com (F.D.); 18943417506@163.com (J.L.)

* Correspondence: liuch0530@163.com (C.L.); liyib@126.com (Y.L.)

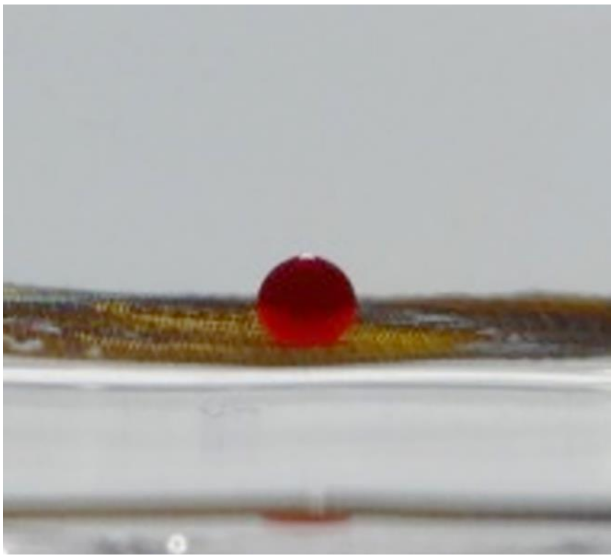

Figure S1. Optical image of an oil droplet (1,2-dichloroethane, dyed by Oil red O) on the BC hydrogel-coated super-wetting membrane surface.

The Optical image of an oil (4 μL, 1,2-dichloroethane) was shown in Figure S1. Moreover, the underwater oil contact angle of the BC hydrogel-coated super-wetting membrane surface was 152.7°, which demonstrated remarkable underwater superoleophobicity of the super-wetting membrane.

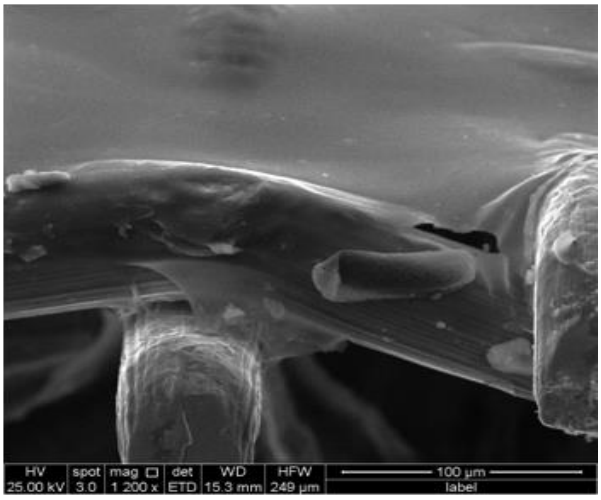

Figure S2. Cross-section SEM images of the BC hydrogel-coated super-wetting membrane.

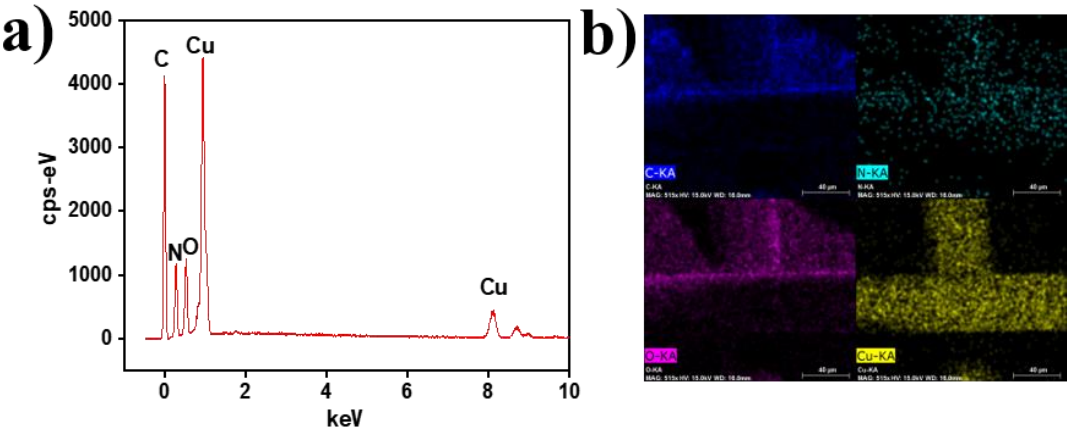

Figure S3. The EDS and elemental analysis of the BC hydrogel-coated super-wetting membrane, (**a**) the chart of element relative content, (**b**) images of surface element distribution.

The BC hydrogel-coated super-wetting membrane surface chemical compositions were further investigated through the energy dispersive spectroscope (EDS) (Figure S3.a-b). The EDS result revealed obvious characteristic peaks in a carbon element, nitrogen element and oxygen element, demonstrating the BC hydrogel has effectively coated the copper mesh.

Movie S1

Recording the water/hexane (dyed by Oil red O) separation process of the BC hydrogel-coated super-wetting membrane through a camera.

Movie S2

Recording the measuring process of the intrusion pressure of the hexane (dyed by Oil red O) on the BC hydrogel-coated super-wetting membrane by a camera.

Movie S3

Recording the rapid departure process of an oil droplet (1,2-dichloroethane) on the BC hydrogel-coated super-wetting membrane surface with a tilt angle of 5.3° through a camera.
